# Ancient Regulatory Role of Lysine Acetylation in Central Metabolism

**DOI:** 10.1128/mBio.01894-17

**Published:** 2017-11-28

**Authors:** Ernesto S. Nakayasu, Meagan C. Burnet, Hanna E. Walukiewicz, Christopher S. Wilkins, Anil K. Shukla, Shelby Brooks, Matthew J. Plutz, Brady D. Lee, Birgit Schilling, Alan J. Wolfe, Susanne Müller, John R. Kirby, Christopher V. Rao, John R. Cort, Samuel H. Payne

**Affiliations:** a Biological Sciences Division, Pacific Northwest National Laboratory, Richland, Washington, USA; b Department of Chemical and Biomolecular Engineering, University of Illinois at Urbana-Champaign, Urbana, Illinois, USA; c Earth Systems Science Division, Pacific Northwest National Laboratory, Richland, Washington, USA; d Buck Institute for Research on Aging, Novato, California, USA; e Department of Microbiology and Immunology, Stritch School of Medicine, Health Sciences Division, Loyola University Chicago, Maywood, Illinois, USA; f Department of Microbiology and Immunology, Medical College of Wisconsin, Milwaukee, Wisconsin, USA; Institut Pasteur

**Keywords:** acetylphosphate, central metabolism, enolase, protein acetylation, proteomics

## Abstract

Lysine acetylation is a common protein post-translational modification in bacteria and eukaryotes. Unlike phosphorylation, whose functional role in signaling has been established, it is unclear what regulatory mechanism acetylation plays and whether it is conserved across evolution. By performing a proteomic analysis of 48 phylogenetically distant bacteria, we discovered conserved acetylation sites on catalytically essential lysine residues that are invariant throughout evolution. Lysine acetylation removes the residue’s charge and changes the shape of the pocket required for substrate or cofactor binding. Two-thirds of glycolytic and tricarboxylic acid (TCA) cycle enzymes are acetylated at these critical sites. Our data suggest that acetylation may play a direct role in metabolic regulation by switching off enzyme activity. We propose that protein acetylation is an ancient and widespread mechanism of protein activity regulation.

## IMPORTANCE

Post-translational modifications can regulate the activity and localization of proteins inside the cell. Similar to phosphorylation, lysine acetylation is present in both eukaryotes and prokaryotes and modifies hundreds to thousands of proteins in cells. However, how lysine acetylation regulates protein function and whether such a mechanism is evolutionarily conserved is still poorly understood. Here, we investigated evolutionary and functional aspects of lysine acetylation by searching for acetylated lysines in a comprehensive proteomic data set from 48 phylogenetically distant bacteria. We found that lysine acetylation occurs in evolutionarily conserved lysine residues in catalytic sites of enzymes involved in central carbon metabolism. Moreover, this modification inhibits enzymatic activity. Our observations suggest that lysine acetylation is an evolutionarily conserved mechanism of controlling central metabolic activity by directly blocking enzyme active sites.

## INTRODUCTION

Post-translational modifications (PTMs) play a critical role in regulating cellular function and response to external stimuli. PTMs, which include phosphorylation, glycosylation, and acetylation, are found in all cell types and lineages and thus likely arose early in evolution. Advances in mass spectrometry-based proteomics have greatly expanded the number of proteins that are known to be modified, but our ability to assign a functional role to specific modifications has lagged. Although acetylation has a well-known role in chromatin remodeling (1), it is now clear that thousands of non-histone proteins are acetylated and that the functional roles of the overwhelming majority of these modifications are unknown. Global lysine acetylation surveys show an enrichment of modified proteins in metabolic pathways in both eukaryotes and bacteria (2, 3), with the most highly enriched pathways being glycolysis and the tricarboxylic acid (TCA) cycle, or citric acid cycle. Even though most proteins in these pathways are multiply acetylated, little is known about the functional consequence of acetylation, including those that might have regulatory activity. For two proteins, previous work has shown that phosphoglycerate mutase (GpmA) and acetyl coenzyme A (acetyl-CoA) synthetase (Acs) both contain lysine residues within the catalytic active site, that acetylation of these residues abolishes enzyme function, and that subsequent deacetylation restores activity (4–6). Although these studies hint at a function for lysine acetylation, it is unclear whether this is an evolutionarily conserved and widespread mechanism.

In this paper, we investigated whether lysine acetylation is found on different enzymes and whether this modification is conserved throughout bacterial evolution by performing a comprehensive phyloproteomic analysis. We found that acetylation occurs in conserved lysine residues located in catalytic pockets of enzymes from the glycolytic pathway and the TCA cycle. Moreover, those modifications substantially changed the physical-chemical properties of the enzyme catalytic pocket inhibiting their activity.

## RESULTS

### Lysine acetylation is a conserved post-translational modification of bacterial proteins.

Our goal was to understand the functional impact of lysine acetylation on proteins and how this was conserved across evolution. Therefore, we first investigated whether acetylation occurs on lysine residues that are conserved in different bacteria. We performed an extensive proteomic analysis of 48 phylogenetically diverse bacteria from six phyla: *Proteobacteria*, *Firmicutes*, *Bacteroidetes*, *Actinobacteria*, *Cyanobacteria*, and *Fibrobacteres*. Each bacterial strain was grown to early stationary phase (for details concerning growth conditions, see Table S1 in the supplemental material) and harvested by centrifugation. The cell pellets were lysed and digested with trypsin. The resultant peptides were then analyzed by liquid chromatography-tandem mass spectrometry. The global proteome coverage for these 48 organisms totaled 73,656 proteins (902,937 peptides) with an average protein false-discovery rate (FDR) of <0.001 (Table S2). Despite not specifically enriching for acetylated peptides prior to the mass spectrometry analysis, we obtained a comprehensive coverage of the bacterial acetylome. A total of 9,107 acetylated proteins, averaging ~190 per organism (24,397 total acetylated peptides, ~508 per organism) were identified. These numbers are substantially larger than those of several previous studies combined together and summarized in a recent review article (7).

10.1128/mBio.01894-17.1 TABLE S1  Information on bacterial strains and growth conditions. Each organism included in the analysis is given, with full strain information where possible and source. Various growth conditions for each sample are also listed. Download TABLE S1, XLS file, 0.05 MB. Copyright © 2017 Nakayasu et al. 2017 Nakayasu et al. This content is distributed under the terms of the Creative Commons Attribution 4.0 International license.

10.1128/mBio.01894-17.2 TABLE S2  Coverage of the phyloproteomic analysis. For each organism, proteomic coverage is reported, listing the number of total peptides and proteins, as well as the acetylated peptides and proteins. The FDR list is also listed, calculated as described in Materials and Methods. Download TABLE S2, XLS file, 0.04 MB. Copyright © 2017 Nakayasu et al. 2017 Nakayasu et al. This content is distributed under the terms of the Creative Commons Attribution 4.0 International license.

**FIG 1:**
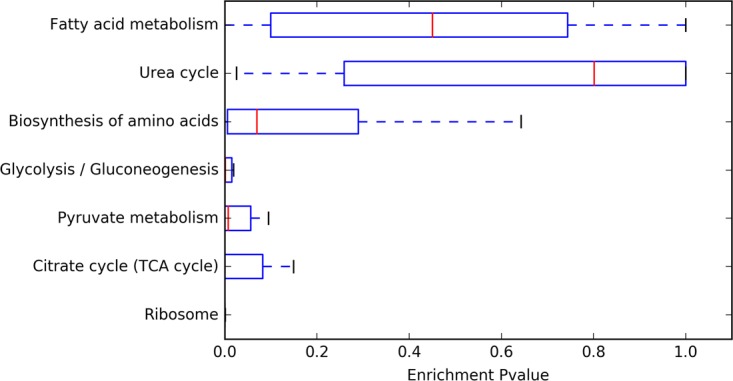
Analysis of pathways enriched in lysine-acetylated proteins. For each organism, we determined whether the set of acetylated proteins was enriched in a given KEGG pathway, calculating the enrichment with a Fisher exact test. The graph shows the average enrichment *P* values across the 48 different bacterial strains.

To study pathways where acetylation is conserved through evolution, we performed a function-enrichment analysis based on the annotations from the Kyoto Encyclopedia of Genes and Genomes (KEGG) database. Acetylated proteins were significantly enriched in glycolysis, pyruvate metabolism, ribosomes, and the TCA cycle, with median enrichment *P* values of <0.002 across organisms (Fig. 1). Although other metabolic pathways in some bacteria were enriched in acetylated proteins (fatty acid metabolism, urea cycle, and biosynthesis of amino acids), the enrichment was inconsistent when considering the broad scope of the phyloproteomic data set. Taken together, these results show that lysine acetylation consistently occurs in proteins involved in glycolysis, pyruvate metabolism, ribosomes, and the TCA cycle, suggesting a common regulatory function in evolutionarily diverse bacteria.

### Enzymes of the glycolytic pathway and the TCA cycle are acetylated in evolutionarily conserved residues of their catalytic pockets.

**FIG 2:**
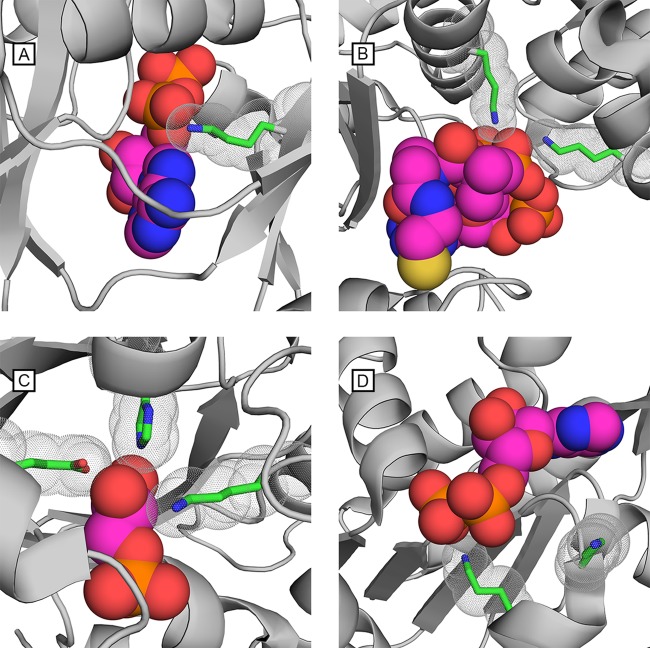
Substrate-binding lysine residues in metabolic enzymes are evolutionarily conserved and acetylated in different organisms. In many glycolytic and TCA cycle catalytic binding sites, lysine is present and universally conserved. The binding pockets of four enzymes are shown with their native substrate or cofactor. Catalytically required lysine residues are shown and outlined with spheres to show their proximity to the substrate or cofactor. (A) Succinyl-CoA synthetase from *E. coli*, PDB 1CQI, shown with the ADP cofactor and lysine 46. (B) Phosphotransacetylase from *Methanosarcina thermophila*, PDB 2AF4, shown with coenzyme A and two lysine residues 257 and 283. (C) Triosephosphate isomerase from *Staphylococcus aureus*, PDB 3UWU, shown with glycerol-3-phosphate and lysine 12. Catalytically essential histidine and glutamic acid residues are also shown. (D) Phosphoglycerate kinase from *Francisella tularensis*, PDB 4FEY, shown with the ADP cofactor and lysine residues 193 and 197.

**FIG 3:**
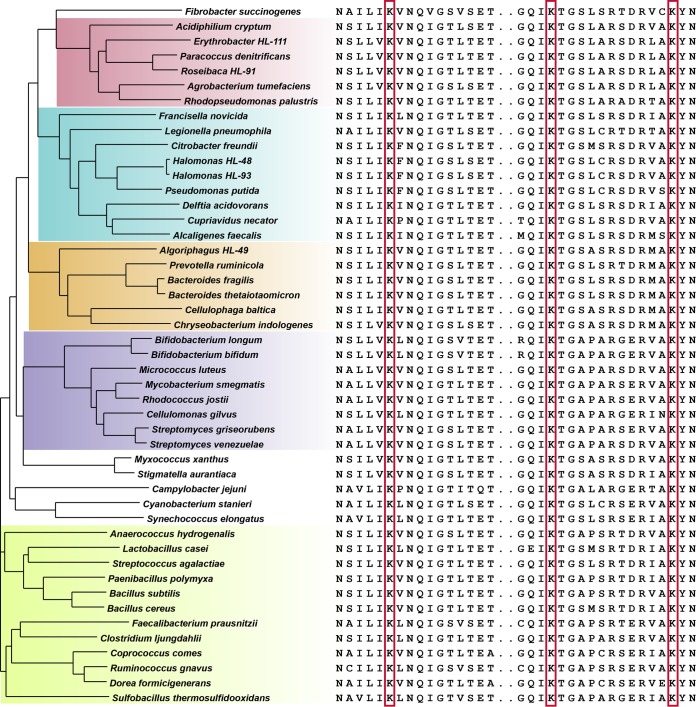
Lysine conservation in enolase across bacteria. A multiple-sequence alignment of enolase showing residues N334 to T349 and G387 to N404 (*B. subtilis*; P37869). Phylogenetic clusters are shown in color to highlight the prominent taxonomic divisions as follows: pink for *Alphaproteobacteria*; blue for *Gamma*- and *Betaproteobacteria*; orange for *Bacteroidetes*; purple for *Actinobacteria*; green for *Firmicutes*. The lysine residues boxed in red are invariant across all organisms.

**FIG 4:**
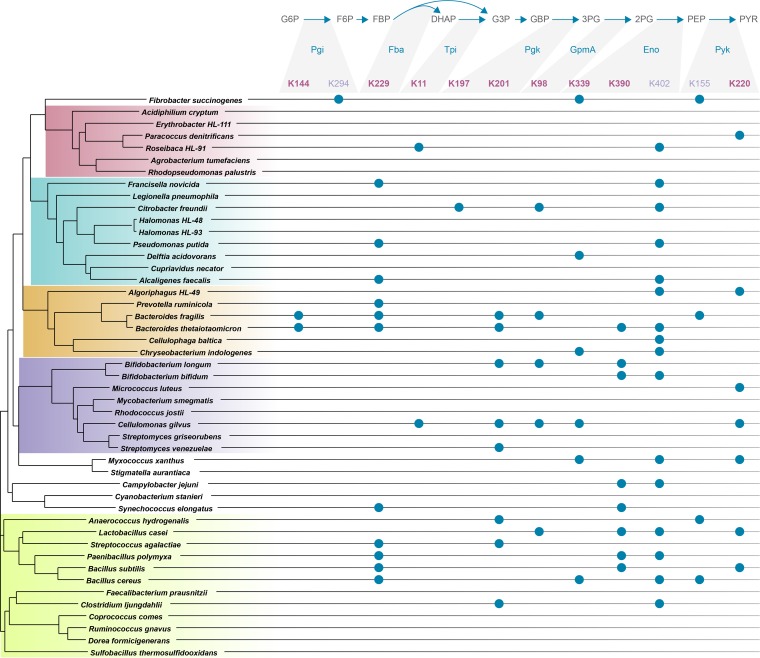
Acetylation of substrate/cofactor-binding lysine residues across taxa. The phyloproteomic data identify acetylated lysines from 48 organisms. Enzymes from glycolysis are listed along with universally conserved lysine residues, numbered according to the numbering system for *Bacillus subtilis* (except for GpmA from *L. casei* gene S6CBB3). Catalytically essential sites as described in the text are shown in boldface type. Nonboldfaced sites are listed if they are universally conserved across bacteria but are not known to be involved in substrate/cofactor binding. Observed acetylations are indicated with solid blue circles. The phylogenetic tree of organisms is based on RplB sequence alignment with the major taxonomic groups colored: *Alphaproteobacteria*, *Gamma*- or *Betaproteobacteria*, *Bacteroidetes*, *Actinobacteria*, and *Firmicutes*. Protein name abbreviations: Pgi, phosphoglucose isomerase; Fba, fructose-bisphosphate aldolase; Tpi, triose-phosphate isomerase; Pgk, phosphoglycerate kinase; GmpA, 2,3-bisphosphoglycerate-dependent phosphoglycerate mutase; Eno, enolase; Pyk, pyruvate kinase.

Due to our interest in the mechanism of enzymatic regulation by acetylation, we narrowed our investigation to specific sites of acetylation and in particular those that are conserved across evolution. We focused on the set of enzymes involved in glycolysis and the TCA cycle and asked whether these enzymes contained lysines involved in substrate or cofactor binding and whether those lysines were identified as acetylated in diverse taxa, potentially pointing to a general regulatory mechanism common to all organisms. In glycolysis, there are nine enzymatic reactions for the conversion of glucose-6-phosphate to pyruvate. Seven of the nine enzymes contain catalytically important lysine residues in the active site (Fig. 2). In four of these enzymes, these lysines bind the substrate (Pgi, Tpi, GpmA, and Eno). In the remaining three enzymes (Fba, Pgk, and Pyk), the active site lysine(s) binds a cofactor, e.g., ATP. All seven enzymes have crystallographic structures that locate the lysine(s) at the binding site (8–14), and protein sequence alignments show that all these lysines are invariant across the 48 bacteria in our phyloproteomic survey (Fig. 3 and supplemental material). Acetylation sites identified in our phyloproteomic data set show that all of the catalytically implicated lysines in these seven glycolytic enzymes are acetylated in multiple organisms (Fig. 4).

We further investigated TCA cycle enzymes for catalytically essential and acetylated lysines. The transition from glycolysis (pyruvate) to the TCA cycle (acetyl-CoA) (part of the pyruvate metabolism pathway) involves a set of four enzymes that control the formation and therefore the pool of available acetyl-CoA. Three of the four enzymes contain lysine residues that participate in catalysis through substrate/cofactor binding. In both Acs (15) and phosphotransacetylase (Pta) (16), the lysine is used to bind the substrate, and in dihydrolipoamide dehydrogenase (17), a subunit of the pyruvate dehydrogenase complex, the lysine binds the flavin adenine dinucleotide (FAD) cofactor. Of the eight enzymatic reactions in the TCA cycle, four contain a catalytic lysine: isocitrate dehydrogenase (18), the alpha-ketoglutarate dehydrogenase complex (17), succinate-CoA ligase (19), and fumarate hydratase (20). With only one exception (fumarate hydratase), these lysines are acetylated in multiple organisms across the phyloproteomic data (see Table S3 in the supplemental material). Summarizing the results, the phyloproteomic analysis quickly identified lysines that are invariant across evolution, participate in catalysis, and are acetylated in multiple organisms; therefore, these acetylations have significant potential to regulate pathway activity. Indeed, this analysis showed that lysine acetylation occurs in catalytically important residues of two-thirds of the enzymes from glycolysis and the TCA cycle. Moreover, in three enzymes, we found additional conserved lysine residues that are acetylated across phyla (Fig. 4, Pgi, Eno, and Pyk). Although these lysines are not currently implicated in substrate/cofactor binding, their conservation across the wide diversity of bacteria suggests that they might be important for protein function.

10.1128/mBio.01894-17.3 TABLE S3  Lysine acetylation on conserved residues in enzyme catalytic regions. For enzymes in the glycolytic and TCA pathways, conserved sites of acetylation are listed. Each site is described by the gene, KEGG ortholog, protein accession number of one reference organism, acetylated sites, and the surrounding protein sequence. Acetylated sites are highlighted in yellow if they are known to bind substrate/cofactors. Download TABLE S3, XLS file, 0.03 MB. Copyright © 2017 Nakayasu et al. 2017 Nakayasu et al. This content is distributed under the terms of the Creative Commons Attribution 4.0 International license.

### Lysine acetylation sterically blocks catalytic pockets and inhibits enzymatic activity.

**FIG 5:**
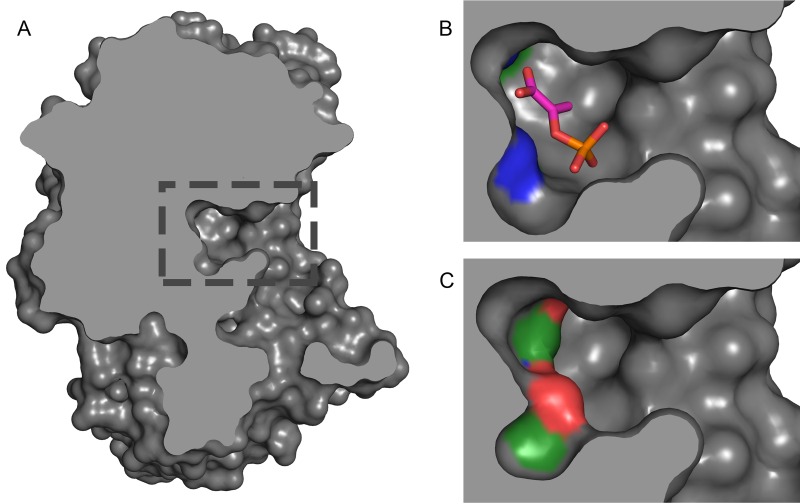
Structural change of enolase acetylation. (A) The catalytic binding site of *Synechococcus elongatus* enolase, PDB 5J04. The boxed region in panel A is enlarged in panels B and C. (B) The binding pocket utilizes two lysine residues (shown in blue at the top and bottom left of the pocket) to bind and stabilize phosphoenolpyruvate (shown as a stick diagram). (C) Acetylating the two active site lysines disrupts both the electrostatic binding potential and the geometry of the binding site, precluding substrate binding.

After finding that lysine acetylation occurs in evolutionarily conserved residues of the catalytic sites of enzymes, we asked whether this modification would regulate enzymatic activity. Therefore, we investigated potential structural and physical/chemical alterations of enzymes due to acetylation. Acetylation on lysine dramatically alters the charge and shape of the lysine residue by neutralizing its positive charge and increasing its size. These alterations change the binding potential and are expected to inhibit catalytic activity. We investigated the impact of acetylation for enolase, which has two substrate-binding lysine residues, by in silico modeling the addition of acetylation on the enzyme structure. Using the crystal structure of *Synechococcus elongatus* enolase (Protein Data Bank [PDB] accession number 5J04) which contains the native substrate (phosphoenolpyruvate [PEP]), the two active site lysines are 2.4 and 2.8 Å from oxygen atoms in the substrate (see Materials and Methods). The volume of the empty active site pocket was 162 Å^3^; with bound PEP, the volume was 70.2 Å^3^. After removing PEP and building acetyl groups on the K339 and K390 side chain amines, the volume of the pocket was 78.7 Å^3^. Thus, the acetylated lysines and the native substrate occupy a similar volume and position within the deeply buried active site (Fig. 5) and the presence of one or both acetyl groups are likely to preclude PEP binding (see Materials and Methods).

**FIG 6:**
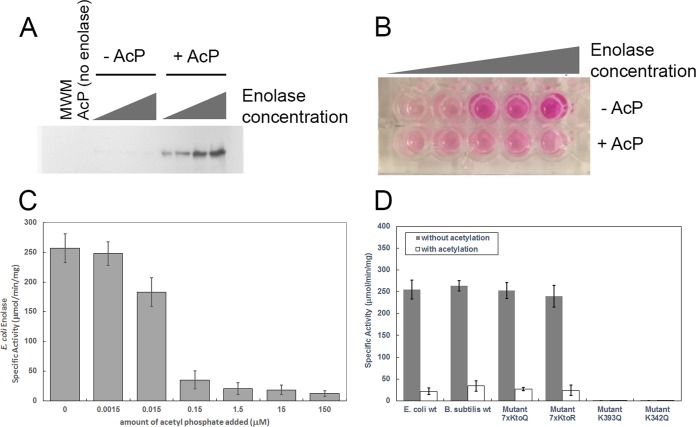
Enolase activity regulation by acetylation. (A) Western blot analysis of enolase treated with acetylphosphate (AcP). From right to left, serial 10 × dilutions of enolase incubated in the presence (+) or absence (−) of 15 µM acetylphosphate. MWM, molecular weight markers. (B) Colorimetric assay of serial 10× dilutions of enolase incubated in the presence or absence of 15 µM acetylphosphate. (C) Enzymatic activity assay for *E. coli* enolase with and without addition of acetyl phosphate. Acetyl phosphate was added at concentrations ranging from 1.5 nM to 150 μM. Acetylation of *E. coli* enolase by acetyl phosphate resulted in a dose-dependent inhibition of *E. coli* enolase activity. (D) Enzymatic activity assay showing that acetylation inhibits enolase catalytic activity. The assay was performed in triplicate. Enolase activity was measured in the presence or absence of 1.5 μM acetylphosphate. Along with wild-type *E. coli* and *B. subtilis* enzymes, four *E. coli* mutant strains were tested. In the 7xKtoQ mutant and 7xKtoR mutant, the seven lysine residues that are not conserved or at the active site were replaced with either glutamine (K→Q) or arginine (K→R) (see Materials and Methods). In the K393Q mutant and K342Q mutant, either active site lysine 393 or active site lysine 342 were mutated to glutamine (see Materials and Methods). wt, wild type.

To test this prediction that acetylation inhibits enzymatic activity and check the evolutionarily conserved nature of this regulatory mechanism, we expressed and measured the catalytic activity of recombinant enolase from both *Escherichia coli* and *Bacillus subtilis*, two phylogenetically distant bacteria. Enzymes were pretreated with acetylphosphate to induce lysine acetylation and tested for activity by monitoring an intermediate generated during the conversion of 2-phosphoglycerate to phosphoenolpyruvate that reacts with a peroxide substrate and generates a colorimetric product. We used a concentration of 15 µM acetylphosphate, which is much lower than physiological conditions. It has been reported that acetylphosphate is present in *E*. *coli* in high micromolar to low millimolar range (21–23). As expected, the acetylphosphate treatment led to a robust acetylation of enolase (Fig. 6A). Confirming our hypothesis, acetylated enolase from *E. coli* lost catalytic activity (Fig. 6B). To understand the sensitivity of the enzyme to acetylation, we performed a titration where the *in vitro* concentration of acetylphosphate was varied from 1.5 nM to 150 µM. The activity of enolase was strongly inhibited by acetylphosphate, even when present at a concentration of 150 nM, much below physiological levels, showing that acetylphosphate is a potent acetylating agent and enzymatic inhibitor (Fig. 6C).

To further demonstrate the specificity of the site of acetylation, we created a mutant *E. coli* enolase where 7 of 10 lysines were mutated to glutamine. Only the two active site lysines and the invariant K405 (UniProt accession number P0A6P9) were kept; all other nonconserved lysines were permuted (Fig. 6D). This mutant was catalytically active and as expected lost catalytic activity when acetylated. A similar mutant was created in which these seven lysine residues were changed to arginine; this mutant also lost catalytic activity when acetylated with 1.5 μM acetylphosphate. Next, we created two mutant enolase enzymes where one of the two catalytic lysines was mutated, either K342→Q or K363→Q. Both of these mutants were catalytically dead, with or without acetylation (Fig. 6D). We also tested the ability of acetylation to inhibit enolase from *B. subtilis*, a phylogenetically distant bacterium, and similar inhibition was observed (Fig. 6D). These results show that lysine acetylation on enzyme active sites substantially modify the catalytic pocket inhibiting the enzymatic activity and that this mechanism is conserved in phylogenetically distant bacteria.

## DISCUSSION

Our investigation shows that 67% of glycolytic and TCA cycle enzymes can be impacted by acetylation of highly conserved lysine residues located within an enzyme’s catalytic pocket. To identify potential regulatory signals for these acetylations, we compared our list of conserved acetyl-lysines to *E. coli* acetylation sites whose relative abundance increased significantly as a function of glucose supplementation to buffered tryptone broth (24). We identified six *E. coli* proteins (TpiA, Pgk, PgmA, Eno, LpdA, and Icd) with invariant lysines within their substrate binding site, whose acetylation was conserved and significantly upregulated in the presence of supplemented glucose (see Table S4 in the supplemental material). Acetyl-lysines in TpiA, GpmA, and Icd are also dependent on acetylphosphate (25), a high-energy acetyl donor that accumulates when carbon flux through glycolysis exceeds the metabolic capacity of the TCA cycle (26). Taken together, these observations suggest the distinct possibility that lysine acetylation functions as a negative-feedback regulatory mechanism to limit metabolism of excess carbon. Because many of the acetylated lysines are located deep within active sites, we presume the acetyl donor for these lysines is a small molecule, such as acetylphosphate (21), and that the reaction may not require an acetyltransferase.

10.1128/mBio.01894-17.4 TABLE S4  Comparison of acetylation sites found in this work with two prior studies of acetylation in *E. coli*. Download TABLE S4, XLS file, 0.02 MB. Copyright © 2017 Nakayasu et al. 2017 Nakayasu et al. This content is distributed under the terms of the Creative Commons Attribution 4.0 International license.

This pattern of active site acetyl-lysines in diverse taxa spans the radiation of bacterial phyla and thus has been conserved through 3.2 billion years of protein sequence divergence. To determine whether the invariant lysines on these metabolic enzymes were conserved outside bacteria, we examined their orthologs in humans and the archaeal *Haloferax volcanii*. With one exception (GpmA in *H. volcanii*), the lysine residues were conserved, suggesting that the use of lysine acetylation as a mechanism to control central metabolic activity is an ancient trait. We also note that acetylphosphate has been proposed to be the most primitive acetyl donor (before evolution of coenzyme A) and likely would have been present at the outset of the evolution of fundamental pathways, such as glycolysis and the TCA cycle (27).

### Conclusions.

In contrast to work that identifies an allosteric regulatory function for post-translational modifications, we discovered that a widespread functional consequence of lysine acetylation is directly inhibiting enzymatic function by modifying amino acid residues involved in catalysis. Although phosphorylation sites used for signaling have been shown to evolve rapidly (28), these lysines are invariant in all bacterial, eukaryotic, and archaeal orthologs. We posit that this regulatory mechanism was established before the last universal common ancestor and represents an ancient and universal functional consequence of lysine acetylation.

## MATERIALS AND METHODS

### Bacterial growth and sample preparation.

Bacteria were grown in 5 ml of medium to early stationary phase. The specific growth conditions used for each of the 48 organisms, including temperature, media, and atmosphere, and source are listed in Table S1 in the supplemental material. Cells were harvested by centrifuging at 3,500 × *g* for 5 min at room temperature. Cell pellets were washed twice by adding 5 ml phosphate-buffered saline (PBS) and centrifuging under the same conditions. Cells were resuspended with 200 μl of 100 mM NH_4_HCO_3_ and lysed by a Bullet Blender (Next Advance) for 4 min at speed 8 in the presence of approximately 100 μl of 0.1-mm zirconia-silica beads at 4°C. Cell lysates were collected into fresh tubes, while the beads were washed with 200 μl of 100 mM NH_4_HCO_3_, and the supernatants were pooled together. The protein concentration was quantified by bicinchoninic acid (BCA) assay (Thermo Fisher Scientific, San Jose, CA), and aliquots of 300 μg of proteins were denatured and reduced by adding 8 M urea and 5 mM dithiothreitol (DTT) and incubating at 60°C for 30 min with shaking at 850 rpm. The reaction mixtures were then diluted 10-fold in 100 mM NH_4_HCO_3_, and 1 M CaCl_2_ was added to a final concentration of 1 mM. Protein digestion was carried out for 3 h at 37°C with a 1/50 trypsin-protein ratio. Digested peptides were desalted in 50-mg C18 cartridges (Strata; Phenomenex) as previously described (29) and quantified by BCA assay before being analyzed by liquid chromatography-tandem mass spectrometry (LC-MS/MS). Samples were divided into blocks and randomized prior to data acquisition on the LC-MS/MS system.

### LC-MS/MS data acquisition.

LC-MS/MS data acquisition was performed as previously described in detail (30). Briefly, peptides were resuspended in water, and a total of 500 ng was loaded into a trap column (5 cm by 360-µm-outer-diameter [OD] by 150-µm-inner-diameter [ID] fused silica capillary tubing [Polymicro, Phoenix, AZ]) packed with 3.6-µm Aeries C18 particles (Phenomenex, Torrance, CA). Separation was performed in a capillary column (70 cm by 360-µm OD by 75-µm ID) packed with 3-µm Jupiter C18 stationary phase (Phenomenex) with a 100-min gradient of acetonitrile (ACN) in water containing 0.1% formic acid. Eluted peptides were analyzed online in a quadrupole-Orbitrap mass spectrometer (Q-Exactive Plus; Thermo Fisher Scientific, San Jose, CA). Tandem mass spectra were collected for the top 12 most intense ions. All data are available as described below in the “Data availability” section.

### Data analysis.

All raw and processed data are available at the PRIDE (*pr*oteomics *ide*ntifications) database (see “Data availability”). Raw mass spectrometry files were converted to the PSI open format mzML (31) using msConvert (32). Files were recalibrated with the mzRefinery tool (33) and searched against species-specific sequence databases using MS-GF+. The search parameters and values or settings were as follows: PrecursorMassTolerance, 20.0 ppm; dynamic modification, acetylation of lysine; maximum modifications per peptide, 3; IsotopeError, −1,1; TargetDecoyAnalysis, true; FragmentationMethod, as written in the spectrum; Instrument, QExactive; Enzyme, Tryp; NumTolerableTermini, 1; MinPeptideLength, 6; MaxPeptideLength, 50; and NumMatchesPerSpec, 1.

All peptide spectrum matches were filtered for false-discovery rate using *q* < 0.001. Protein identifications required two peptides. Protein functional annotation was derived using the KEGG automated annotation, GhostKoala (34). Figure 1 and Table S2 in the supplemental material present the pathway enrichment and peptide/protein statistics and were derived from these data using an iPython notebook, publicly available at https://github.com/samuelpayne/Biodiversity.Acetylation.Supplement.Coverage. Pathway enrichment, as encoded in the notebook, was performed using a Fisher exact test with functional annotations from KEGG and the background proteome as proteins identified by MS/MS but not annotated as being part of the pathway (see iPython notebook for details). We tracked the functional enrichment for all pathways for all organisms. The data presented are the median enrichment of pathways across all organisms.

Final annotation for multiple-sequence alignments was hand curated to remove weak scoring paralogs and proteins with alternate annotation (e.g., at UniProt or RefSeq). Multiple-sequence alignments were performed using Muscle (35) via a web application programming interface (API) (36), and peptide identifications were mapped visually onto multiple-sequence alignments using the peptide homology viewer (37). The phylogenetic tree which is part of Fig. 3 and 4 was generated from a multiple-sequence alignment of ribosomal protein large subunit 2 (RplB) from each organism in the library; graphical representation of the tree was generated using an iPython notebook, publicly available at https://github.com/samuelpayne/TreeFigureForBiodiversityLibrary, utilizing the ETE 3 toolkit for phylogenomic data (38). This tree was stylized in Adobe Illustrator and appended with the multiple-sequence alignment (Fig. 3) or the acetylation sites (Fig. 4). The annotated multiple-sequence alignment for all pathway enzymes is provided as html files with full sequence alignments noting observed peptides and acetylated lysines; these files are located at https://github.com/samuelpayne/TreeFigureForBiodiversityLibrary.

Protein structures from the Protein Data Bank (PDB) were identified via their UniProt accession number or manual search for a protein’s functional annotation using the https://www.wwpdb.org/ website. The structures visualized in Fig. 2 and 5 were created using the PyMol software (http://www.pymol.org). The enolase active site (PDB 5J04 structure) was analyzed with NACCESS 2.1.1 (39) and CASTp (40) to gauge accessibility of acetylated lysines and measure the volume of the active site pocket. We found accessibilities relative to the tripeptide Ala-Lys-Ala to be 12.9 to 13.4% for side chain atoms for K339 and 5.7 to 6.0% for side chain atoms for K390 (ranges reflect different polypeptides in the asymmetric unit). Other lysines in the PDB 5J04 structure have relative side chain atom accessibilities ranging from 0.6% to 94%. The distance from K390 Nε to phosphoenolpyruvate (PEP) O1′ (one of the carboxylate O) is 2.4 Å, while the distance from K339 Nε to PEP O2P (one of the phosphate O) is 2.8 Å. The volume of the empty active site pocket in the PDB 5J04 structure was 162 Å^3^; with bound PEP, the volume was 70.2 Å^3^. After removing PEP and building acetyl groups on the K339 and K390 side chain amines, the volume of the pocket was 78.7 Å^3^.

Mapping the sites of acetylated lysines to the data from Schilling et al. (24) was manually done for each protein using MUSCLE, and the proteins were listed in Table S4 of reference 24. The following sites were found to correspond to the invariant lysines. For GpmA, K98 from *Lactobacillus casei* maps to K100 of *E. coli*. For Eno, K339 from *B. subtilis* maps to K342 of *E. coli*. For Pgk, K201 of *B. subtilis* maps to K197 of *E. coli*. For LpdA, K56 of *B. subtilis* maps to K54 of *E. coli*. For Tpi, K11 of *B. subtilis* maps to K11 of *E. coli*. For Icd, K226 of *B. subtilis* maps to K235 of *E. coli*. Similar mapping was done for the sites identified by Kuhn et al. (25).

### Cloning and purification of enolase.

Both the *E. coli* and *B. subtilis* enolase were constructed such that they contained an amino-terminal 6×His tag. The *E. coli* enolase was PCR amplified from *E. coli* DH5α using primers MP042 (GTC GGC TAG CTC CAA AAT CGT AAA AAT CAT CGG TC) and MP043 (TGA CAA GCT TAG ATA GCG GCG GAT TTA GC). These primers were designed to remove the start codon and add a 5′ NheI site and a 3′ HindIII site. The *B. subtilis* enolase was PCR amplified from *B. subtilis* strain OI3269 using primers MP071 (GTC GGC TAG CCC ATA CAT TGT TGA TGT TTA TGC AC) and MP072 (TGA CCT CGA GGG TAA GGC TTT ATT TGG ATC TCT G). These primers were designed to remove the start codon and add a 5′ NheI site and a 3′ XhoI site. The mutant *E. coli* 7xKtoQ enolase was made by ordering a gBlock from IDT (sequence below) in which lysines at positions 6, 62, 82, 85, 257, 328, and 419 were mutated to glutamines by changing the codons from AAA to CAA. The gBlock contained a 5′ NheI site and a 3′ HindIII site. The respective DNA fragment and the pET28a plasmid were then digested using the respective restriction enzymes, gel purified, and ligated together. The resulting plasmid constructs were then transformed in *E. coli* BL21(DE3).

The mutant *E. coli* 7xKtoQ enolase sequence (gBlock) follows (restriction sites and mutations are shown by lowercase letters): GTCGgctagcTCCAAAATCGTAcaaATCATCGGTCGTGAAATCATCGACTCCCGTGGTAACCCGACTGTTGAAGCCGAAGTACATCTGGAGGGTGGTTTCGTCGGTATGGCAGCTGCTCCGTCAGGTGCTTCTACTGGTTCCCGTGAAGCTCTGGAACTGCGCGATGGCGACAAATCCCGTTTCCTGGGTcaaGGCGTAACCAAAGCTGTTGCTGCGGTAAACGGCCCGATCGCTCAGGCGCTGATTGGCcaaGATGCTcaaGATCAGGCTGGCATTGACAAGATCATGATCGACCTGGACGGCACCGAAAACAAATCCAAATTCGGCGCGAACGCAATCCTGGCTGTATCTCTGGCTAACGCCAAAGCTGCTGCAGCTGCTAAAGGTATGCCGCTGTACGAGCACATCGCTGAACTGAACGGTACTCCGGGCAAATACTCTATGCCGGTTCCGATGATGAACATCATCAACGGTGGTGAGCACGCTGACAACAACGTTGATATCCAGGAATTCATGATTCAGCCGGTTGGCGCGAAAACTGTGAAAGAAGCCATCCGCATGGGTTCTGAAGTTTTCCATCACCTGGCAAAAGTTCTGAAAGCGAAAGGCATGAACACTGCTGTTGGTGACGAAGGTGGCTATGCGCCGAACCTGGGTTCCAACGCTGAAGCTCTGGCTGTTATCGCTGAAGCTGTTAAAGCTGCTGGTTATGAACTGGGCAAAGACATCACTTTGGCGATGGACTGCGCAGCTTCTGAATTCTACAAAGATGGTcaaTACGTTCTGGCTGGCGAAGGCAACAAAGCGTTCACCTCTGAAGAATTCACTCACTTCCTGGAAGAACTGACCAAACAGTACCCGATCGTTTCTATCGAAGACGGTCTGGACGAATCTGACTGGGACGGTTTCGCATACCAGACCAAAGTTCTGGGCGACAAAATCCAGCTGGTTGGTGACGACCTGTTCGTAACCAACACCAAGATCCTGcaaGAAGGTATCGAAAAAGGTATCGCTAACTCCATCCTGATCAAATTCAACCAGATCGGTTCTCTGACCGAAACTCTGGCTGCAATCAAGATGGCGAAAGATGCTGGCTACACTGCAGTTATCTCTCACCGTTCTGGCGAAACTGAAGACGCTACCATCGCTGACCTGGCTGTTGGTACTGCTGCAGGCCAGATCAAAACTGGTTCTATGAGCCGTTCTGACCGTGTTGCTAAATACAACCAGCTGATTCGTATCGAAGAAGCTCTGGGCGAAcaaGCACCGTACAACGGTCGTAAAGAGATCAAAGGCCAGGCATAAaagcttGTCA.

All enolase constructs were purified on a HisTrap column (5 ml; GE Healthcare) on an ÄKTA pure chromatography system (GE Healthcare) using the denaturing protocol provided by the manufacturer. Fractions were collected and run on an SDS-polyacrylamide gel to check for the elution of the desired 6×His fusion protein. The pure-protein flowthrough was collected and dialyzed against four changes of 1 liter of TKMD (50 mM Tris [pH 8.0], 50 mM KCl, 5 mM MgCl_2_, 0.1 mM dithiothreitol [DTT], 10% [vol/vol] glycerol) and then stored at −80°C.

### Enolase acetylation and activity.

Purified enolase protein at a concentration of 1.25 mM was dissolved in 150 mM Tris HCl (pH 7.3), 10% glycerol, 10 mM MgCl_2_, and 150 mM NaCl. Enolase was acetylated by adding acetyl phosphate (see the legend to Fig. 6 for concentrations) and incubating for 1 h at 37°C. Acetylated and nonacetylated samples were then immediately used to measure enolase activity as described below, or the reactions were stopped by the addition of 2× SDS loading buffer (120 mM Tris HCl [pH 6.8], 4% SDS, 0.2% bromophenol blue, 20% glycerol, and 10% 2-mercaptoethanol). In the latter case, samples were loaded onto an SDS-polyacylamide gel and subjected to Western blotting according to a protocol previously described (41). Acetylation of enolase was verified by probing the Western blot using an anti-acetylated lysine primary antibody (Cell Signaling Technology) diluted 1,000×, followed by a horseradish peroxidase (HRP)-conjugated anti-rabbit antibody (Cell Signaling) diluted 2,000×. The blot was developed with a chemiluminescence assay (ECL Plus; GE Healthcare) following the manufacturer recommendations.

Enolase activity was measured using an enolase activity assay kit (Sigma-Aldrich). Briefly, this kit works via a coupled enzyme assay in which d-2-phosphoglycerate is converted to PEP, resulting in the formation of an intermediate that reacts with a peroxidase substrate, generating a colorimetric (570-nm) product proportional to the enolase activity present. The assay was repeated on three separate days using four dilutions of each enolase construct in acetylated and nonacetylated form to determine enolase activity.

### Code availability.

All data processing tools are publicly available at https://github.com/ or https://sourceforge.net/. Data format conversion and calibration were performed using msconvert, part of the proteowizard project (source code at http://proteowizard.sourceforge.net/). MSGF+ was used to annotate tandem mass spectral identification (source code at https://github.com/sangtaekim/msgfplus and webserver at https://massive.ucsd.edu/ProteoSAFe/static/massive.jsp). Postprocessing scripts as described above are available at https://github.com/samuelpayne/Biodiversity.Acetylation.Supplement.Coverage and https://github.com/samuelpayne/TreeFigureForBiodiversityLibrary.

### Data availability.

All primary mass spectrometry files and identification results are freely available at the proteomic data repository PRIDE under accession no. PXD005851.
